# Bibliometric analysis of librarian involvement in systematic reviews at the University of Alberta

**DOI:** 10.29173/jchla29696

**Published:** 2024-04-01

**Authors:** Megan R. Kennedy, Janice Y. Kung

**Affiliations:** Geoffrey and Robyn Sperber Health Sciences Library, University of Alberta Library, Edmonton, AB

## Abstract

**Introduction:**

It is well documented that librarian involvement in systematic reviews generally increases quality of reporting and the review overall. We used bibliometric analysis methods to analyze the level of librarian involvement in systematic reviews conducted at the University of Alberta (U of A).

**Methods:**

Using Web of Science (WoS), we searched for systematic reviews completed in the years 2016-2020 with a U of A co-author. Systematic reviews identified through WoS were screened in two phases: (*i*) exclusion of duplicates, protocols, other types of reviews, and systematic review methodology literature to leave true systematic review publications, and (*ii*) screening for level of librarian involvement (acknowledgement, co-author, or no involvement).

**Results:**

640 reviews were analyzed for the following categories: (*i*) librarian named as a coauthor; (*ii*) librarian named in the acknowledgements section; (*iii*) librarian mentioned in the body of the manuscript; (*iv*) no librarian involvement. We identified 152 reviews who named a librarian as a co-author on the paper, 125 reviews named a librarian in the acknowledgements section, and 67 reviews mentioned a librarian in the body of the review without naming them as a co-author or in an acknowledgement. WoS Research Areas were used to identify disciplines that used librarian support and those that did not. A keyword network analysis revealed research areas that were very active in producing systematic reviews, while also providing information on the areas publishing systematic reviews without librarian support.

**Conclusion:**

There is a great deal of variation in how the work of librarians is reflected in systematic reviews. This was particularly apparent in reviews where a librarian was mentioned in the body of the review but they were not named as an author or formally acknowledged. Continuing to educate researchers about the work of librarians is crucial to fully represent the value librarians bring to systematic reviews. Bibliometric analysis provides useful insights on service gaps for specific disciplines or research areas that are currently not using librarian support in systematic review publications, which can help inform service planning.

## Introduction

"Despite the fact that systematic review searching is a significant task for many librarians and knowledge professionals, the search process can be considered a form of invisible labor because it often goes without recognition" [1]. As we move into an increasingly metrics focused world, it is more important than ever for librarians and their institutions to be able to provide tangible ways of expressing how effectively they spend their time. This is important on a personal level for librarians who may be going through the tenure and promotion process to advance their careers. As well, it is important to understand research output on an institutional level to properly plan and distribute resources to meet the needs of users, including identifying any service gaps that may exist within an institution. With this in mind, we initiated this project with the intention to learn more about the state of systematic review publishing at the University of Alberta and to understand the extent of librarian involvement in these reviews.

The systematic review is a common research methodology employed in health sciences disciplines, and increasingly in other non-health disciplines as well. The goal of a systematic review is to identify and synthesize all possible sources of evidence that are appropriate and relevant to a given research question.

Thorough identification of sources is a vital element of completing a high-quality systematic review that requires the very high-level expert searching skills of a librarian or information specialist. Many sources of methodological guidance, including the Cochrane Handbook of Systematic Reviews of Interventions, and many funding agencies, including the Institute of Medicine (IOM) of the National Academy of Science’s Standards for Systematic Reviews and the Canadian Institutes of Health Research (CIHR), recommend or require that a librarian be involved in the search process for a systematic review. Further, Rethlefsen et al. published PRISMA-S, an extension to the Preferred Reporting Items for Systematic Reviews and Meta-Analyses (PRISMA), in 2021 [2]. This extension focuses on search strategy reporting and suggests that an obstacle to high quality reporting in systematic reviews is “that many systematic reviews do not include librarians or information specialists as members of the systematic review team or as authors on the final manuscript” [2]. Librarian involvement can help to ensure searches performed are high quality and well documented so that the review is more transparent and reproducible. Librarian involvement can also help to reduce possible risk of bias. Several studies demonstrate how librarians as co-authors in systematic reviews is correlated with a higher quality in review reporting, particularly the search methodology [3–7]. Rethlefsen et al. also suggest that should a research team choose not to work directly with a librarian when conducting their systematic searches, they strongly encourage “having the search strategy peer reviewed by an experienced searcher, information specialist, or librarian” [2]. However, studies also show that the work of librarians is poorly reported in systematic reviews [1,6]. Similar to a study conducted by Ross-White in 2016 [8], we wanted to know if the advice from systematic review publishing agencies and guidelines, such as PRISMA-S, to utilize librarians in the systematic review process was translating to reality at the University of Alberta and to what extent librarians were contributing to these reviews.

Similar to the 2016 study by Ross-White conducted at Queen's University and the 2020 update to this environmental scan [8,9], we aimed to analyze whether librarians receive co-authorship or other acknowledgments for their work on systematic reviews. We undertook a bibliometric analysis of systematic reviews published by University of Alberta researchers with the aim to identify the level of librarian involvement in these reviews. Were they recognized (*i*) as authors; (*ii*) in formal acknowledgements (i.e., named in the acknowledgement section of a paper); (*iii*) in informal acknowledgements (i.e., named in the body of the manuscript, such as in the methods section); or (*iv*) not recognized at all? Bibliometrics is defined as: “the analysis of published information (usually books, journal articles, datasets, etc.) and its related metadata (for example, abstracts, authors, keywords, citations) using statistics to describe or show relationships between published works” [10]. Moreover, “bibliometrics is based on the assumption that a field’s scholarly output is captured in the published literature” [11]. This is an effective analytical approach to explore and analyze large volumes of data and identify trends in the literature [12]. Bibliometric analysis fits the needs of this project as we wanted to quantify the variable of librarian involvement in published systematic reviews and also identify other characteristics of these reviews, including journal impact factors, journal titles, and research areas.

This study aims to use bibliometric analysis as evidence to inform health librarianship practice at one research-intensive university. We will accomplish this in two ways: (*i*) determine to what extent librarians are directly and indirectly involved in systematic review publications through co-authorships or acknowledgements; and (*ii*) identify the range of systematic review publications by discipline and topic to recognize outreach initiatives for areas where librarians are not as heavily involved.

## Methods

The initial step of this analysis involved identifying relevant literature for analysis. We carried out a search on November 4, 2021 using Web of Science Core Collection to capture systematic reviews published between 2016-2020 by University of Alberta researchers. The rationale behind choosing the five-year data range was to ensure the dataset included five full calendar years’ worth of publications to provide a sample of the publications from one academic institution. We opted to use Web of Science Core Collection (including the three main Citation indexes: Sciences (SCI, IC, CCR), Social Sciences (SSCI), Arts & Humanities (A&HCI)) to complete this step because of the built-in bibliometric tools of this database, such as the feature to easily identify journal impact factors of relevant reviews, which would be useful later in the analysis (see Appendix - Full search strategy and database information). The search was conducted by searching for “systematic review” in the title field and “University of Alberta” in the affiliation field. Results were limited to publication date 2016-2020.

As is good practice in bibliometric analyses, we opted to use a single data source in order to draw more accurate comparisons of the data in our analysis [13]. While there are other types of evidence synthesis reviews that may involve librarians, systematic reviews are one of the more methodologically established review types. Systematic reviews of effectiveness of interventions in health and healthcare have been around since the late 1970's and early 1980's and the Cochrane Collaboration and Campbell Collaboration, leaders in methodological guidance for systematic reviews, both began back in the 1990's. PRISMA [14], a core reporting guideline that includes a 27-item checklist for increased clarity, transparency, and quality in reporting of systematic reviews, was first published in 2009. Comparably, the earliest methodological guidance for scoping reviews did not come until 2005 with Arksey and O'Malley [15], and this was a limited guidance document that spoke more so to the theory behind scoping reviews, rather than how to conduct and report them. Further, PRISMA-ScR [16], the reporting guideline extension for scoping reviews, was only published in 2018. Similar patterns are present for other review types including rapid reviews, umbrella reviews, realist reviews, evidence gap maps, etc. Given the greater history of the systematic review methodology, the scope of this analysis is limited to systematic review publications. We identified 772 records in total and began our first of two screening phases. Records were imported into Endnote for deduplication. The primary phase of screening was conducted in Excel and eliminated the following records: (*i*) duplicates; (*ii*) systematic review protocols; (*iii*) other kinds of reviews, such as scoping reviews; (*iv*) methodological papers. We decided to remove systematic review protocols from our analysis since we believed these types of records would be less likely to involve a librarian (in comparison to their full-scale review counterparts). This decision was made based on our experiences as health librarians supporting systematic reviews. In our second screening phase, we retrieved the full-text documents of the records and eliminated any conference materials or other abstract-only materials, as well as records where no full-text was available electronically.

**Fig. 1:**
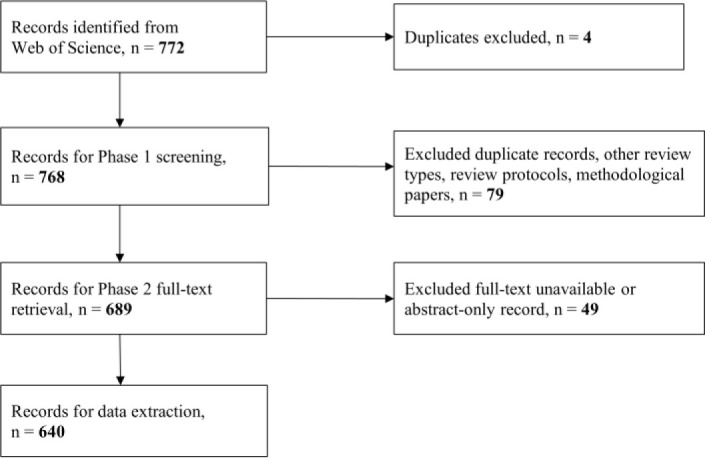
Flow-chart of record identification and screening

During data extraction, we coded reviews according to the level of librarian involvement and used Excel to record the data. We classified the extent of librarian involvement into four categories: (*i*) librarian recognized as an author; (*ii*) librarian recognized in the acknowledgement section of the paper (formal acknowledgement); (*iii*) librarian recognized in the body of the paper, such as in the methods section (informal acknowledgement); (*iv*) no librarian involved or recognized on the review. Librarians were identified by position title (e.g., librarian or information specialist), name of library in author information (e.g. John W. Scott Health Sciences Library), or academic credentials (e.g., MLIS or similar degree). We extracted additional information, specifically librarian affiliation, if we found there was librarian involvement as coauthor or formal acknowledgement in the review. If there was no formal librarian involvement, we scanned the full-text for references to librarian or information specialist contributions in the review such as in the Methods section (e.g., “We consulted a librarian for assistance in the search strategy”). Figure 1 outlines the flow of information through the different phases of the project.

We conducted a bibliometric analysis on citations, compiled the most popular journals (and their journal impact factors), as well as a keyword network analysis of included papers to identify the disciplines and research areas producing systematic review publications, and the areas where there was more or less librarian involvement. The bibliometric analysis was accomplished by taking the Digital Object Identifiers of included studies and searching for the articles once again in Web of Science Core Collection to export a clean dataset of systematic review publications that met the inclusion criteria of the study. This plain text file was subsequently imported into VOSviewer for further analysis. The data that support the findings of this study are available on request.

## Results

We conducted two different analyses with the 640 papers included in the analysis: one with librarian involvement and one with no formal librarian involvement.

### Formal librarian involvement

**Fig. 2:**
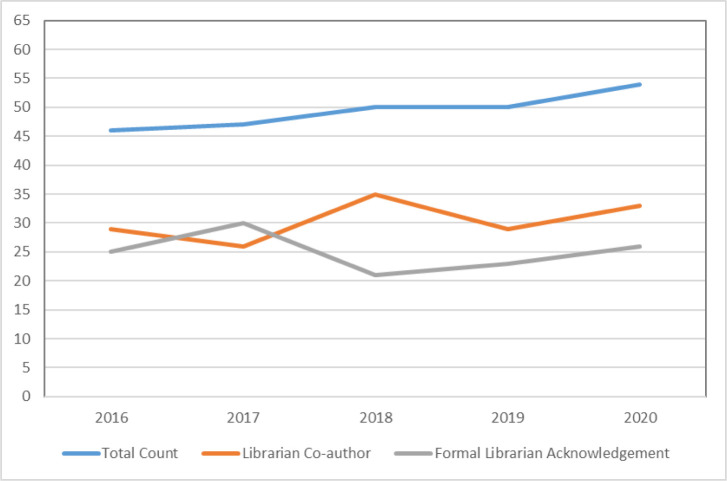
Graphical representation of librarian involvement (data from Table 1)

Of the 640 papers, 247 (39%) had some form of formal librarian involvement, which included librarians listed as co-authors or formally recognized for their contributions in the acknowledgement section of the publication. Table 1 summarizes the number of publications broken down by year and displayed graphically in Figure 2. There were 152 papers (24%) with librarian co-authors and 125 publications (20%) that acknowledged librarians. There are some instances where a paper had both a librarian co-author and a second different librarian in the acknowledgements section (e.g., another librarian who performed the Peer Review of Electronic Search Strategies (PRESS)). By breaking down the co-authorship papers even further, 62 (10%) papers included librarians with affiliations to the John W. Scott Health Sciences Library and 90 (14%) librarian co-authors were from other institutions or University of Alberta departments outside of the Library.

**Table 1:** Systematic reviews published with formal librarian involvement

Year	Total Count	Librarian Co-author	Formal Librarian Acknowledgement
2016	46	29	25
2017	47	26	30
2018	50	35	21
2019	50	29	23
2020	54	33	26

**Fig. 3:**
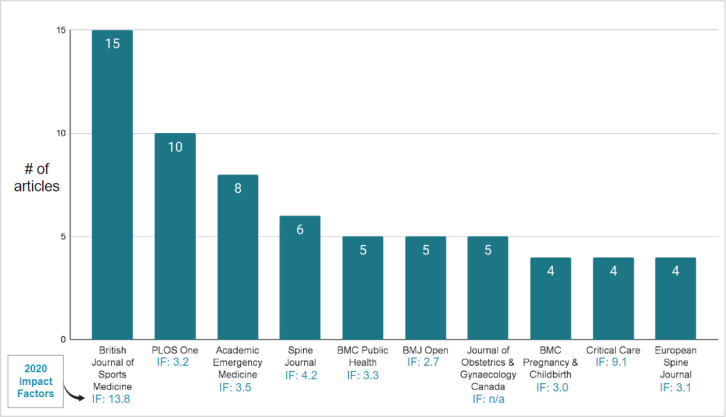
Top journals with librarian involvement

Web of Science assigns research areas to journals, a schema comprising approximately 250 subject areas across many different disciplines [17]. Journals may be categorized under several subject areas and because of this, the frequency of publications results in higher numbers than the number of actual publications in the dataset. For example, *Spine Journal* falls under two categories: Orthopedics and Clinical Neurology. Within the five-year timeframe (2016-2020), the research area with the highest frequency of publications was Health Care Sciences Services (n=85), followed by Pediatrics (n=74), General Internal Medicine (n=62), Pathology (n=62), Neurosciences Neurology (n=59), Psychology (n=52), Behavioral Sciences (n=49), Pharmacology Pharmacy (n=47), Rehabilitation (n=46), and Orthopedics (n=44). Determining the top journals in which the systematic reviews were published provides useful insights on the disciplinary coverage and journal coverage of the publications that had substantial librarian involvement. This is also indicative of the strong areas of research performed at the University of Alberta. Figure 3 highlights the top ten journals where the systematic reviews were published, including their 2020 journal impact factors. “The Journal Impact Factor is defined as all citations to the journal in the current JCR year to items published in the previous two years, divided by the total number of scholarly items (these comprise articles, reviews, and proceedings papers) published in the journal in the previous two years" [18]. Reviewing journal impact factors offers some insights on the perceived quality of where authors are publishing. For many authors, it is generally assumed that if an article is published in a journal with “high impact,” the article is of high scientific quality. However, this metric is not a full representation of quality and this will be explored further in the Discussion section. The *British Journal of Sports Medicine* had the highest frequency with 15 systematic review publications in the included studies, which implies that librarians are indeed involved in systematic review publications considered to be high quality.

### No formal librarian involvement

On the other hand, 393 (61%) papers had no formal librarian involvement. Of the 393 reviews with no librarian co-author or formal librarian acknowledgement, 67 publications (17%) mentioned some form of librarian consultation or assistance in the methods section of the full-text. Authors used language such as, “The search strategy was designed with the assistance of a librarian specialized in health sciences,” “With a medical librarian, we did a comprehensive systematic search,” or “An information specialist did a comprehensive literature search.”

For papers with no formal librarians involved, the Web of Science Research Areas with the highest frequency were Pharmacology Pharmacy (n=90), Dentistry Oral Surgery Medicine (n=86), Health Care Sciences Services (n=86), Surgery (n=78), Cardiovascular System Cardiology (n=73), Neurosciences Neurology (n=72), Behavioral Sciences (n=69), Nutrition Dietetics (n=67), Pathology (n=67), and Psychology (n=58). Interestingly, there is some subject area overlap with publications including librarian co-authors or acknowledgements. In fact, out of the top ten Web of Science Research Areas covered, half of the subject areas have both librarian involvement and no librarian involvement; they include Pharmacology Pharmacy, Health Care Sciences Services, Neurosciences Neurology, Behavioral Sciences, and Psychology.

### Keyword network analysis

All keywords from the 640 systematic review articles were analyzed to better understand the types of research conducted based on the frequency count of author-supplied keywords, how they are interconnected, and their citation impact. VOSviewer (version 1.6.18) was used to create the following network maps based upon keywords (noun phrases) indexed in Web of Science Core Collection. After removing “systematic review” as a keyword, we developed a co-occurrence analysis of the remaining author keywords that appeared at least three times. 117 out of the 1558 total keywords in the dataset met this threshold but only 108 keywords were linked with the corpus and ultimately used in the visualizations. The keyword frequency count, appearing a minimum of three times, and the selection of the 108 keywords were system-generated by VOSviewer by default. Cooccurrence analysis is a visualization strategy of counting paired terms or phrases that frequently appear together, to demonstrate their connectedness. If we did not remove “systematic review” from the analysis, the strongest co-occurrence in this dataset would have been between the terms “systematic review” and “meta-analysis.” To take it one step further, VOSviewer groups keywords that commonly appear together into clusters.

**Fig. 4:**
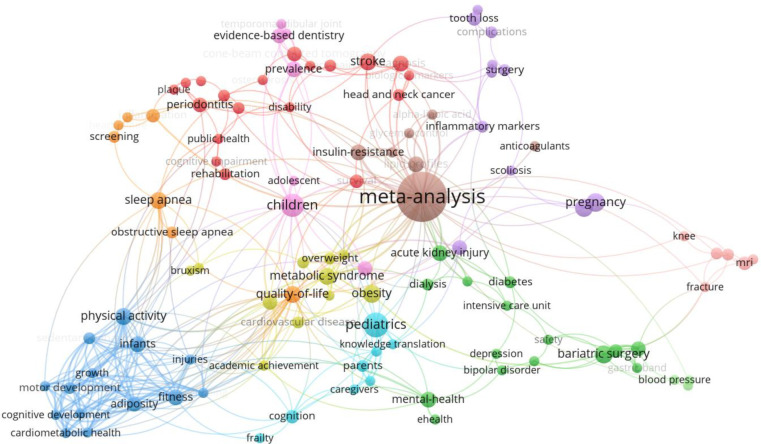
Network analysis by author keyword for all 640 papers

This network analysis identified 11 clusters represented by different colours in Figure 4. The keywords are represented by the nodes or circles in the visualization. The sizes of the labels and circles are determined by the weight of the items. The higher the weight, the larger the label and circle of the item. The more central the items appear in the diagram, the more well connected they are to other keywords. Less connected terms appear at the outer edges of the network. The number of lines between nodes also represents the number of connections between nodes.

There are notable clusters (or topics) shown for sleep apnea, physical activity, pediatrics, acute kidney injury, bariatric surgery, pregnancy, and stroke. The network map conveys useful insights on the focus of these papers. For example, the orange cluster of sleep apnea is connected to “screening,” which provides more information on the areas of study concerning sleep apnea. Many systematic review publications also performed meta-analyses, which is the largest node in the network map.

**Fig. 5:**
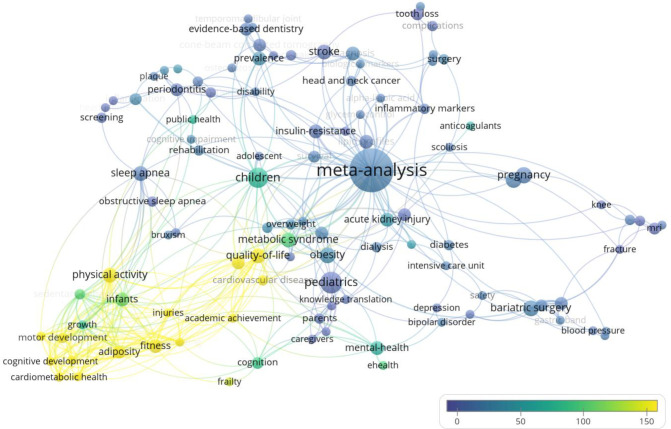
Average number of citations received by keyword (n=640)

The same visualization can be overlaid with citation data to help determine which keywords are associated with higher or lower citation-based impact. In Figure 5, the colour of the node indicates the average number of citations each keyword has received. The colour yellow indicates a higher number of citations, retrieving approximately 150 citations. For example, papers related to physical activity, children, and metabolic syndrome are most highly cited compared to other keywords in this network analysis.

**Fig. 6:**
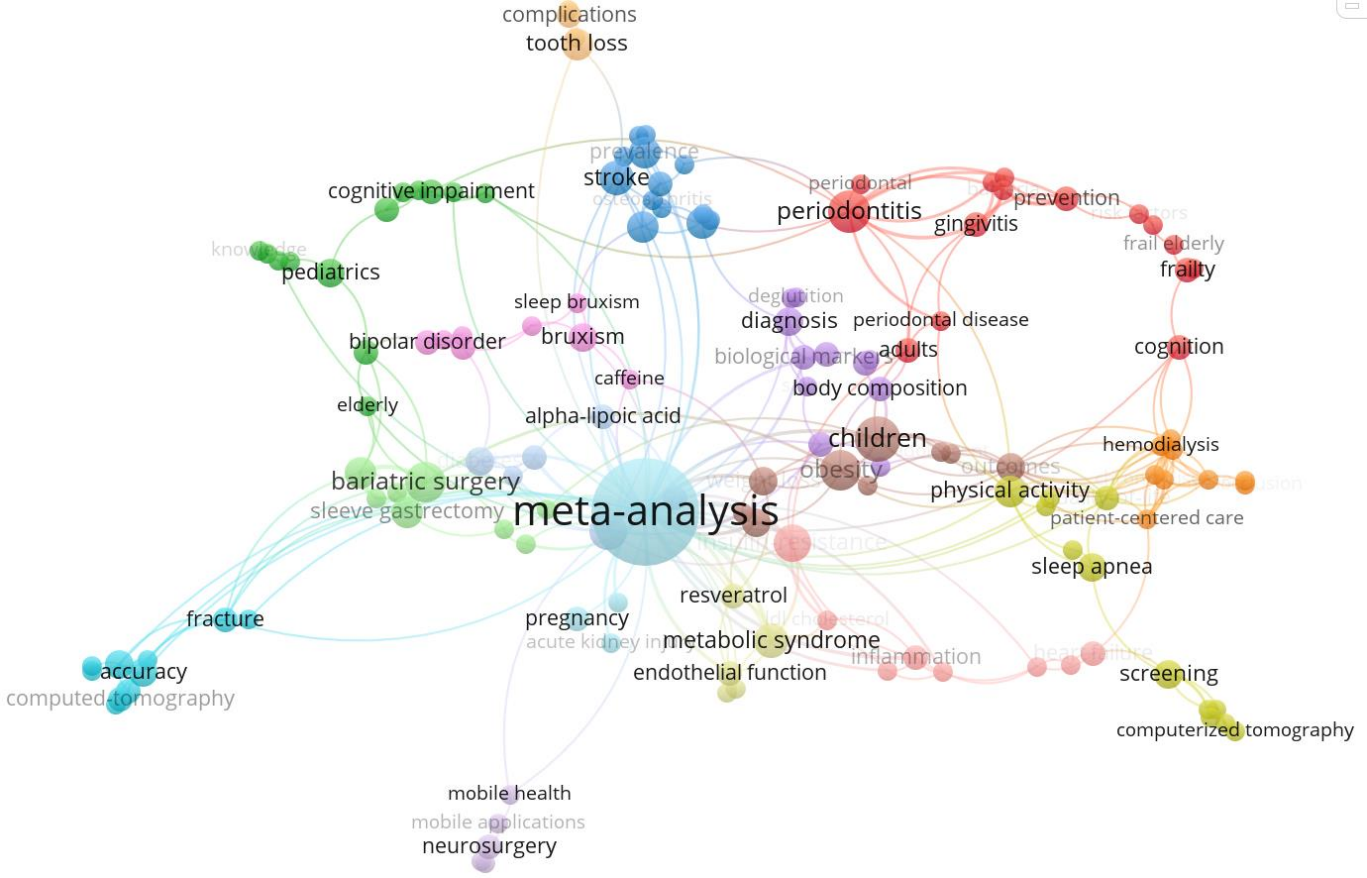
Network analysis by author keyword for papers with no formal librarian involvement (n=393)

For the 393 publications where librarians were not formally involved, a similar level of network analysis was conducted with author-supplied keywords. After removing “systematic review” as a keyword, we developed a co-occurrence analysis of the remaining author keywords that appeared at least two times. 164 out of the 1098 total keywords in the dataset met this threshold but only 140 keywords were linked with the corpus and used in the visualization in Figure 6. Again, the minimum frequency count for keywords (i.e. appearing at least two times) and the number of keywords identified for meeting the threshold were system-generated by VOSviewer by default. The nodes are stronger for keywords including periodontitis (red node), physical activity (yellow), bariatric surgery (green), bruxism (pink), and stroke (dark blue).

**Fig. 7:**
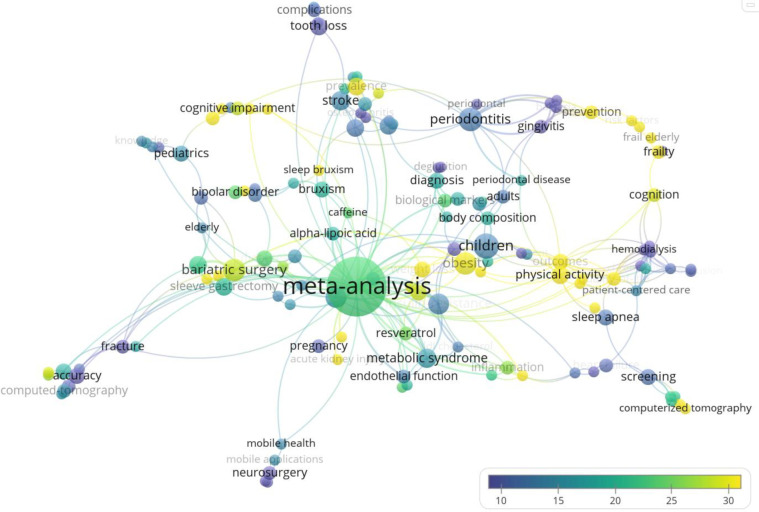
Average number of citations received by keyword for papers with no formal librarian involvement (n=393)

Figure 7 highlights subject areas or topics that had greater impact based on citation counts. The yellow nodes are the most highly cited topics (with approximately 30 citations); they include physical activity, obesity, and computerized tomography.

## Discussion

University of Alberta researchers published at least 640 systematic reviews between 2016-2020 and the number of papers published with librarian involvement remained consistent across the years with 46-54 publications per year. The level of librarian support provided to University of Alberta affiliated members remained consistent. 39% of all publications evaluated had formal librarian involvement either as co-authors or were officially acknowledged. Upon closer inspection, 10% of papers included librarian co-authors with University of Alberta affiliations, particularly with the John W. Scott Health Sciences Library (Scott Library). The staff complement in the Scott Library includes five full-time subject librarians and four cross-appointed librarians who work in research institutes four days a week and dedicate one day a week at Scott Library (0.2 Full-time Equivalent (FTE) per person, or 0.8 FTE total for the four cross-appointed librarians). This provides a snapshot and overview on librarian productivity for published manuscripts in five years at one research-intensive university, which includes a medicine program. Scott Library librarians co-authored 62 publications even though collaborating with researchers on systematic review projects is not a core component of our work. Rather, systematic review collaboration falls under the research portfolio and is optional. Librarians have the autonomy to decide their level of involvement in research projects. While evaluating affiliation records, it was interesting to see that there was inconsistent and improper affiliation attribution to the Scott Library. Examples included Health Sciences Library, Scott Health Sciences Library, 2K4.01 Walter MacKenzie Health Science Center, JWS Health Sciences Library, and Faculty of Nursing. Inconsistent affiliation labels are not unique to libraries; the lack of standardization with university affiliation names is a known issue when it comes to bibliometric work [19], and this issue is also prevalent in department affiliations. The improper affiliation attribution relates to the cases where librarians are affiliated with faculties instead of libraries. One possible reason for this is the limitations imposed by journal websites to select affiliations from a controlled list, and some do not have “health sciences library” as an option. Therefore, librarians get attributed to the faculty level (e.g., Faculty of Nursing) instead of the library. While reviewing the acknowledgement sections, we also observed misspelled names (e.g., Lisa Chan instead of Liza Chan).

Although many standards recommend or require librarian involvement in systematic review projects, including The Cochrane Handbook for Systematic Reviews of Interventions [20], IOM Standards for Systematic Reviews - particularly Standard 3.1 [21], and the CIHR [22], fewer than 50% of the evaluated publications had a librarian formally involved. Based on the findings with low librarian involvement, researchers may not be aware or may not find value for librarians to provide support in systematic review projects. This is a topic worth further exploration. Regardless of the decision on why there are few systematic reviews with librarian involvement, there is potential for growth when it comes to librarian outreach efforts to certain groups. There are faculties on campus that are very active in conducting and publishing systematic reviews compared to others. The dataset shows the continued dominance of health sciences researchers who publish this type of review study over other disciplines. The systematic review research methodology has gained traction in disciplines outside of health sciences such as in business [23], education [24], environmental science [25], social sciences [26], and software engineering [27]. However, there were very few publications outside of health from University of Alberta researchers.

To determine which faculties or subject areas to expand our outreach initiatives into, we may consider five of the top ten Web of Science Research Areas that did not have formal librarian involvement. The five research areas include Dentistry Oral Surgery Medicine, Surgery, Cardiovascular System Cardiology, Pathology, and Nutrition Dietetics. These areas are confirmed with the keyword network analysis (Figures 6 and 7) where there are several systematic review publications related to bariatric surgery and periodontitis as examples. Despite the clear opportunities to expand librarian services and support to researchers, it raises two questions: Are researchers aware that they may involve librarians in these projects? Do librarians have the capacity to take on more work? Several studies found systematic reviews with librarian co-authors had more comprehensive search strategies, were more reproducible, had better documentation, and had lower risk of bias [3,4,7,28]. Comparatively, systematic reviews that only mentioned librarians in passing, acknowledged librarians, or had no librarian involvement were not as comprehensive or as well documented [5]. Since the majority of the 640 studies did not have librarian co-authors or any librarian involvement, this may be an indication that the quality of searches in systematic reviews is not prioritized by publishers or authors and are still getting published.

There may be a number of different reasons and obstacles that prevent librarians from being collaborators, such as training and mentoring, time, value in the context of institutional culture, support from library administrators, and structure of librarian services within an institution [29,30]. Additionally, some research teams may be resistant to add a librarian as a co-author believing that they may not meet the International Committee of Medical Journal Editors' (ICMJE) standards for co-authorship [31–33]. Some researchers misunderstand the role of librarians working on systematic reviews and may see librarians as only doing administrative work (e.g. retrieving PDFs) [33]. Studies suggest that issues related to co-authorship should be addressed and formalized early in the review process. Further, health sciences librarians are not immune to experiencing burnout in the workplace especially with supporting systematic review projects [34]. While being mindful of librarian capacity, library administration could make evidence-informed decisions for recruitment or targeted outreach initiatives to specific departments.

A recent paper by Thelwall et al. suggests that citation counts are an indicator of quality and therefore, a paper with a high citation count could be deemed “high quality” [35]. However, it is established within the bibliometrics literature that bibliometric analysis comes with limitations. While it is understood that citation counts may be used as a proxy to measure research impact based on the influence one paper has by being cited by another [36], it does not take into account the reasons why papers are cited.

## Limitations

We acknowledge that this study is limited by choosing to analyze only systematic reviews, rather than the full spectrum of evidence synthesis review types, such as scoping, rapid, umbrella, and realist reviews. This is an area for further exploration in an additional study. Further, we recognize that improper attribution and misspelled names are known issues in scholarly publishing, and this could affect the total number of librarians identified as authors in our analysis. Unfortunately, there was little that the research team could do to mitigate this issue in the study. Web of Science Core Collection was used as the only data source for identifying University of Alberta systematic review publications to eliminate inconsistent citation records for analysis. However, since the dataset came from a single source, it is possible some studies may have been missed, especially from non-health or science disciplines. However, since systematic reviews are most prolific in the health sciences, Web of Science Core Collection was deemed a reasonable database to use for this study. Further, it is common in bibliographic analysis to select only a single data source - usually Web of Science or Scopus due to the advanced citation metrics features available in these databases. These limitations could be avoided in future studies by including other forms of analytics and data sources such as alternative metrics (views, downloads, social media mentions, patent citations, etc.), or conducting qualitative studies that include surveys to learn why researchers choose not to consult librarians for evidence synthesis projects. The findings demonstrate only systematic review projects that were published but do not cover librarians’ work for unpublished reviews (either abandoned or in-progress), which speaks to the invisible labour librarians perform when they contribute intellectually to such projects [1].

## Conclusion

We conducted a bibliometric analysis to gauge the level of librarian involvement in systematic reviews published by University of Alberta researchers within the time span of 2016-2020. Among 640 included studies, 39% of publications had some form of librarian involvement as co-authors or formally acknowledged. Of those librarian co-author papers, 10% were affiliated with the John W. Scott Health Sciences Library. A keyword network analysis revealed research areas that were very active in producing systematic reviews, while also providing information on the areas publishing systematic reviews without librarian support. This study demonstrates how bibliometric analysis can provide an overview of the systematic review research output from one academic institution and contribute meaningful insights concerning the areas on campus where researchers are actively working on systematic review projects but not consulting librarians. This type of analysis is beneficial to help inform our professional practice by expanding outreach initiatives to faculties and departments.

## Appendix - Full search strategy and database information

### Database Information: Web of Science - Core Collection via Clarivate

#### Date of search: November 4, 2021

- Index Chemicus (IC): 1993 to 2023

- Current Chemical Reactions (CCR): 1985 to 2023

- Science Citation Index (SCI): 1900 to 2023

- Arts & Humanities Citation Index (A&HCI): 1975 to 2023

- Book Citation Index (BKCI): 2005 to 2023

- Emerging Sources Citation Index (ESCI): 2018 to 2023 - Social Sciences Citation Index (SSCI): 1900 to 2023

- Conference Proceedings Citation Index - Science (CPCI–S): 1990 to 2023

- Conference Proceedings Citation Index - Social Sciences & Humanities (CPCI-SSH): 1990 to 2023

#### Search strategy

TI="systematic review" AND OG="University of Alberta"

Limit Date: 2016-2020

## References

[1] Ross-White A. Search is a verb: systematic review searching as invisible labor. J Med Libr Assoc. 2021 Oct 5;109(3):505–9. 34629983 10.5195/jmla.2021.1226 PMC8485967

[2] Rethlefsen ML, Kirtley S, Waffenschmidt S, Ayala AP, Moher D, Page MJ, et al. PRISMAS: an extension to the PRISMA statement for reporting literature searches in systematic reviews. Syst Rev. 2021 Jan 26;10(1):39. 33499930 10.1186/s13643-020-01542-z PMC7839230

[3] Rethlefsen ML, Farrell AM, Osterhaus Trzasko LC, Brigham TJ. Librarian co-authors correlated with higher quality reported search strategies in general internal medicine systematic reviews. J Clin Epidemiol. 2015 Jun;68(6):617–26. 25766056 10.1016/j.jclinepi.2014.11.025

[4] Schellinger J, Sewell K, Bloss JE, Ebron T, Forbes C. The effect of librarian involvement on the quality of systematic reviews in dental medicine. PLOS ONE. 2021 Sep 1;16(9):e0256833. 34469487 10.1371/journal.pone.0256833 PMC8409615

[5] Toews LC. Compliance of systematic reviews in veterinary journals with Preferred Reporting Items for Systematic Reviews and Meta-Analysis (PRISMA) literature search reporting guidelines. J Med Libr Assoc. 2017 Jul 7;105(3):233–9. 28670210 10.5195/jmla.2017.246 PMC5490700

[6] Koffel JB. Use of recommended search strategies in systematic reviews and the impact of librarian involvement: a cross-sectional survey of recent authors. PLOS ONE. 2015 May 4;10(5):e0125931. 25938454 10.1371/journal.pone.0125931 PMC4418838

[7] Meert D, Torabi N, Costella J. Impact of librarians on reporting of the literature searching component of pediatric systematic reviews. J Med Libr Assoc JMLA. 2016 Oct;104(4):267–77. 27822147 10.3163/1536-5050.104.4.004 PMC5079487

[8] Ross-White A. Librarian involvement in systematic reviews at Queen’s University: an environmental scan. J Can Health Libr Assoc J Assoc Bibl Santé Can [Internet]. 2016 Aug 7 [cited 2023 Aug 10];37(2). Available from: https://journals.library.ualberta.ca/jchla/index.php/jchla/article/view/26149. 10.29173/jchla29517 PMC9327591 35949918

[9] Ross-White A. An environmental scan of librarian involvement in systematic reviews at Queen’s University: 2020 update. J Can Health Libr Assoc. 2021 Aug 1;42(2):110–7. 35949918 10.29173/jchla29517 PMC9327591

[10] Broadus RN. Toward a definition of “bibliometrics.” Scientometrics. 1987 Nov 1;12(5):373–9. Available from: 10.1007/BF02016680.

[11] Ninkov A, Frank JR, Maggio LA. Bibliometrics: Methods for studying academic publishing. Perspect Med Educ. 2022 Jun 1;11(3):173–6. 34914027 10.1007/s40037-021-00695-4 PMC9240160

[12] Donthu N, Kumar S, Mukherjee D, Pandey N, Lim WM. How to conduct a bibliometric analysis: An overview and guidelines. J Bus Res. 2021 Sep 1;133:285–96.

[13] University College London (UCL). Library Services. 2018 [cited 2023 Aug 10]. Guidance on using bibliometrics. Available from: https://www.ucl.ac.uk/library/research-support/bibliometrics/guidance-using-bibliometrics.

[14] Page MJ, McKenzie JE, Bossuyt PM, Boutron I, Hoffmann TC, Mulrow CD, et al. The PRISMA 2020 statement: an updated guideline for reporting systematic reviews. BMJ. 2021 Mar 29;372:n71. 33782057 10.1136/bmj.n71 PMC8005924

[15] Arksey H, O’Malley L. Scoping studies: towards a methodological framework. Int J Soc Res Methodol. 2005 Feb 1;8(1):19–32.

[16] Tricco A, Lillie E, Zarin W, O’Brien K, Colquhoun H, Levac D, et al. PRISMA Extension for Scoping Reviews (PRISMAScR): Checklist and Explanation | Annals of Internal Medicine. Ann Intern Medicine. 2018 Oct 2;169(7):467–73. 10.7326/M18-0850 30178033

[17] Web of Science Research Areas [Internet]. [cited 2023 Aug 10]. Available from: https://incites.help.clarivate.com/Content/Research-Areas/wos-research-areashtm

[18] Clarivate JCR (JCR). Glossary. [cited 2023 Nov 1]. Journal Impact Factor. Available from: https://jcr.help.clarivate.com/Content/glossary.htm.

[19] Taşkın Z, Al U. Standardization problem of author affiliations in citation indexes. Scientometrics. 2014 Jan 1;98(1):347–68.

[20] Chapter 4: Searching for and selecting studies [Internet]. [cited 2023 Aug 10]. Available from: https://training.cochrane.org/handbook/current/chapter-04.

[21] Eden J, Levit L, Berg A, Morton S, editors. Finding What Works in Health Care: Standards for Systematic Reviews [Internet]. Washington, D.C.: National Academies Press; 2011 [cited 2023 Aug 10]. Available from: https://www.nap.edu/catalog/13059. 24983062

[22] Canadian Institutes of Health Research. Knowledge Synthesis - Tips for Success - CIHR [Internet]. 2013 [cited 2023 Aug 10]. Available from: https://cihrirsc.gc.ca/e/46891.html.

[23] Savabieh S, Nayebzadeh S, Abghari R, Hatami-Nasab SH. A systematic review and a synthesis research on market orientation studies. Int J Inf Sci Manag IJISM. 2020 Aug 1;18(2):245–61.

[24] Evans J, Benefield P. Systematic reviews of educational research: does the medical model fit? Br Educ Res J. 2001;27(5):527–41.

[25] Bilotta GS, Milner AM, Boyd I. On the use of systematic reviews to inform environmental policies. Environ Sci Policy. 2014 Oct 1;42:67–77.

[26] Petticrew, M, Roberts H. Systematic Reviews in the Social Sciences: A Practical Guide [Internet]. Wiley; 2006. Available from: 10.1002/9780470754887.

[27] Babar MA, Zhang H. Systematic literature reviews in software engineering: preliminary results from interviews with researchers. In: 2009 3rd International Symposium on Empirical Software Engineering and Measurement. 2009. p. 346–55.

[28] Aamodt M, Huurdeman H, Strømme H. Librarian co-authored systematic reviews are associated with lower risk of bias compared to systematic reviews with acknowledgement of librarians or no participation by librarians. Evid Based Libr Inf Pract. 2019 Dec 16;14(4):103–27.

[29] Gore GC, Jones J. Systematic reviews and ribrarians: a primer for managers. Partnersh Can J Libr Inf Pract Res [Internet]. 2015 Jul 10 [cited 2023 Aug 10];10(1). Available from: https://journal.lib.uoguelph.ca/index.php/perj/article/view/3343.

[30] Bloss JE, Sewell K, Schellinger J, Haberstroh A. Health sciences and medical librarians conducting research and their experiences asking for co-authorship. J Med Libr Assoc JMLA. 110(4):449–62. 10.5195/jmla.2022.1485 PMC10124612 37101919

[31] O’Dwyer LC, Wafford QE. Addressing challenges with systematic review teams through effective communication: a case report. J Med Libr Assoc JMLA. 109(4):643–7. 10.5195/jmla.2021.1222 PMC8608185 34858096

[32] International Committee of Medical Journal Editors. Recommendations: Defining the Role of Authors and Contributors [Internet]. [cited 2023 Aug 10]. Available from: https://www.icmje.org/recommendations/browse/roles-and-responsibilities/defining-the-role-of-authors-and-contributors.html.

[33] Nicholson J, McCrillis A, Williams JD. Collaboration challenges in systematic reviews: a survey of health sciences librarians. J Med Libr Assoc JMLA. 2017 Oct;105(4):385–93. 28983202 10.5195/jmla.2017.176 PMC5624428

[34] Demetres MR, Wright DN, DeRosa AP. Burnout among medical and health sciences information professionals who support systematic reviews: an exploratory study. J Med Libr Assoc. 2020 Jan 2;108(1):89–97. 31897056 10.5195/jmla.2020.665 PMC6919998

[35] Thelwall M, Kousha K, Stuart E, Makita M, Abdoli M, Wilson P, et al. In which fields are citations indicators of research quality? J Assoc Inf Sci Technol. 2023 Aug 1;74(8):941–53.

[36] Belter CW. Bibliometric indicators: opportunities and limits. J Med Libr Assoc JMLA. 2015 Oct;103(4):219–21. 26512227 10.3163/1536-5050.103.4.014 PMC4613388

